# Acute Effects of the 5‐Hydroxytryptamine Type 4 Receptor Agonist Mosapride on Pharyngeal Swallowing Physiology in Adults

**DOI:** 10.1002/kjm2.70125

**Published:** 2025-10-09

**Authors:** Shu‐Wei Liang, Jui‐Sheng Hung, Taher Omari, Ming‐Wun Wong, Wei‐Yi Lei, Tso‐Tsai Liu, Chih‐Hsun Yi, Chien‐Lin Chen

**Affiliations:** ^1^ Division of Gastroenterology and Hepatology, Department of Internal Medicine Chung Shan Medical University Hospital Taichung Taiwan; ^2^ Department of Medicine, Hualien Tzu Chi Hospital Buddhist Tzu Chi Medical Foundation and Tzu Chi University Hualien Taiwan; ^3^ Flinders Health and Medical Research Institute Flinders University Adelaide Australia; ^4^ Institute of Medical Sciences Tzu Chi University Hualien Taiwan

**Keywords:** 5‐hydroxytryptamine type 4 receptor agonist, high‐resolution impedance manometry, pharyngeal swallowing

## Abstract

The 5‐hydroxytryptamine type 4 receptor agonist mosapride is known to modulate esophageal peristalsis and enhance lower esophageal sphincter compliance. However, its impact on oropharyngeal swallowing physiology remains insufficiently characterized. This study aimed to investigate the acute effects of mosapride on oropharyngeal swallowing dynamics in adults. Participants received either oral mosapride (40 mg) or placebo 1 h prior to testing in a randomized, crossover design. High‐resolution impedance manometry was performed using a solid‐state catheter to measure pressure and impedance within the oropharyngeal segment. Each participant underwent at least three swallows of 5, 10, and 20 mL thin and thick liquids administered by syringe. Manometric data were analyzed using the Swallow Gateway web‐based platform. Twenty‐four volunteers (13 male, mean age, 33 years; range, 24–56 years) completed the study. During thick swallows, mosapride significantly increased upper esophageal sphincter (UES) maximal opening admittance compared to placebo (5 mL: *p* = 0.017; 10 mL: *p* = 0.008) and reduced UES integrated relaxation pressure (10 mL: *p* = 0.018; 20 mL: *p* = 0.017). No significant effects were observed during thin liquid swallows. A marginal reduction in pre‐deglutitive UES basal pressure was noted during 5 mL thick swallows (*p* = 0.050). Following adjustment using repeated measures ANOVA, only the reduction in UES basal pressure remained statistically significant (*p* = 0.040). Acute administration of oral mosapride results in reduced UES basal tone in adults. These findings provide novel physiological evidence suggesting that mosapride may modulate neuroregulatory mechanisms controlling UES contractility.

## 1 Introduction

Swallowing in the pharynx involves a coordinated sequence consisting of upper esophageal sphincter (UES) relaxation and contraction of the pharyngeal constrictor muscles, allowing the bolus to be transferred into the esophagus. Impairment in UES relaxation or pharyngeal constrictor muscle contraction may result in dysphagia, aspiration pneumonia, and malnutrition. At rest, the UES maintains tonic contraction to prevent the retrograde movement of gastric contents [1].

5‐hydroxytryptamine type 4 (5‐HT4) receptors are present in the central nervous system, particularly enriched in the striatum, substantia nigra, olfactory tubercle, and hippocampus [2]. In the enteric nervous system, 5‐HT4 receptors are also distributed on neurons of the myenteric plexus and on smooth muscle cells [3].

Mosapride, a widely used prokinetic agent in clinical practice, exerts its effect by activating 5‐HT4 receptors to promote acetylcholine release from nerve endings within the myenteric plexus [4]. Manometric studies have demonstrated that mosapride enhances esophageal motility by increasing the amplitude of both primary and secondary peristalsis in the esophageal smooth muscle and elevates lower esophageal sphincter (LES) resting pressure [5, 6].

To date, no published studies have investigated the impact of mosapride on pharyngeal swallowing physiology. Furthermore, there is a lack of direct functional evidence supporting the involvement of 5‐HT4 receptors in central motor neuron activation or in striated muscle contraction. Therefore, the aim of this exploratory study was to evaluate and compare pharyngeal manometric parameters following the administration of mosapride versus placebo.

## 2 Methods

### 2.1 Subjects

This was an open‐label prospective study. Eligible participants were adults aged 20–60 years with no history or symptoms of dysphagia. Subjects were recruited through public advertisements. Exclusion criteria included any history of esophageal stricture, prior gastrointestinal or abdominal surgery, current use of medications known to affect gastrointestinal motility, or the presence of psychiatric disorders.

### 2.2 Study Design

This clinical trial was conducted at Hualien Tzu Chi Hospital, Hualien, Taiwan. Participants were randomized using a computer‐generated sequence to receive either mosapride or placebo prior to the initial high‐resolution pharyngeal manometry examination. In the placebo arm, no active medication was administered. All participants underwent pharyngeal high‐resolution impedance manometry (HRIM) on two separate occasions, with a minimum interval of 2 weeks between assessments. Participants were randomized to receive either mosapride (40 mg) or placebo 1 h prior to the examination, in a crossover design. On each study day, a solid‐state HRIM catheter (Solar GI acquisition unit, Laborie/MMS) was inserted transnasally, and pressure as well as impedance data of the oropharyngeal region were recorded. The study employed an open‐label, randomized crossover design, wherein the order of mosapride and placebo administration was randomized to mitigate potential adaptation effects associated with repeated manometric assessments.

Bolus swallowing was assessed using at least three volumes (5, 10, and 20 mL) of test boluses. Boluses were prepared using saline mixed with a thickening agent (SBMkit, Trisco Foods, Brisbane, Australia) to achieve thin and thick consistencies corresponding to International Dysphagia Diet Standardization Initiative (IDDSI) Levels 0 and 4, respectively. The consistency of the test boluses was verified according to IDDSI flow test standards [7].

As no prior studies have evaluated the effects of mosapride on pharyngeal swallowing physiology, sample size estimation was based on preliminary data from a related investigation involving esophageal manometry [5]. A minimum of 10 subjects was calculated to be sufficient to detect statistically significant differences with 80% power. The study protocol was reviewed and approved by the Institutional Review Board of Hualien Tzu Chi Hospital (IRB No. IRB109‐220‐A).

### 2.3 Manometric Data Analysis

**FIGURE 1:**
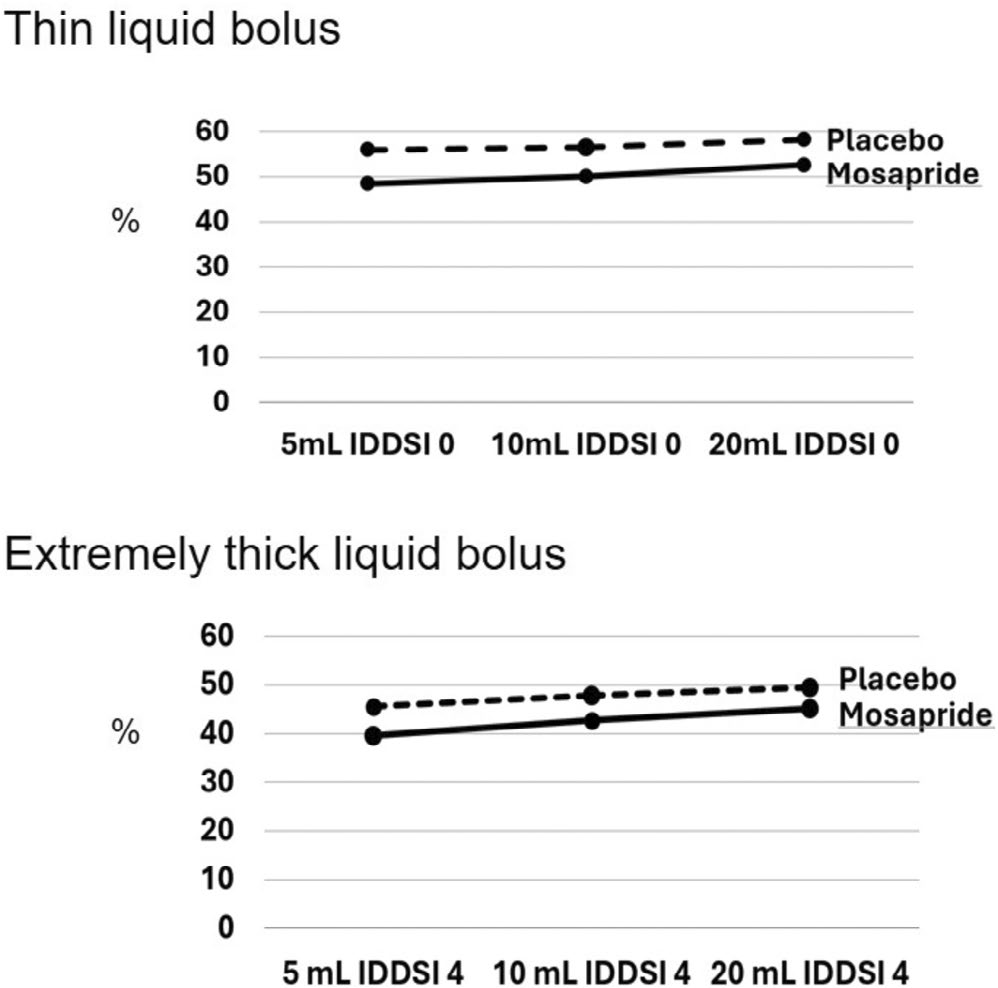
Pre‐deglutitive upper esophageal sphincter (UES) basal pressure across different bolus volumes and viscosities.

**FIGURE 2:**
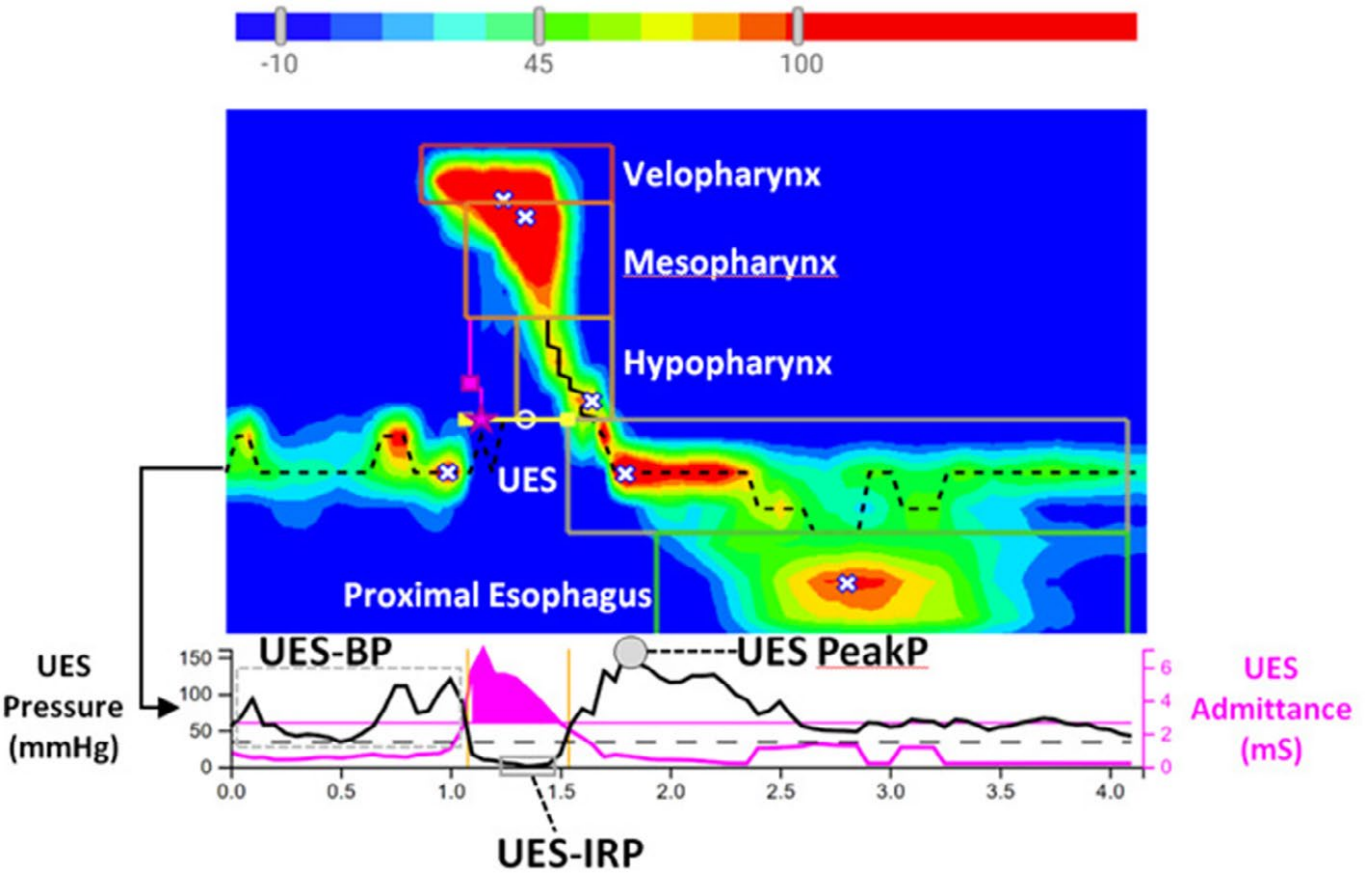
Representative pharyngeal high‐resolution impedance manometry (HRIM) topography and associated swallowing metrics. The middle panel displays a representative pressure topography obtained from pharyngeal high‐resolution impedance manometry (HRIM). The color gradient bar at the top indicates pressure intensity, with blue representing the lowest and red the highest pressure. The pressure wave is segmented into five anatomical components used for calculating contractile integrals: The velopharynx, mesopharynx, hypopharynx, upper esophageal sphincter (UES), and proximal esophagus. The pink asterisk (★) denotes the location of maximum UES distension area admittance, while the pink square (■) indicates the site of intrabolus distension pressure. The lower panel shows the corresponding waveform data, where black represents pressure and pink indicates admittance. The interval between two vertical yellow lines reflects the UES relaxation time (UES RT). HRIM, high‐resolution impedance manometry; UES BP, pre‐deglutitive UES basal pressure; UES PeakP, post‐deglutitive UES peak pressure; UESIRP, UES integrated relaxation pressure.

HRIM data were uploaded and analyzed using the Swallow Gateway web‐based analysis platform (www.swallowgateway.com; Flinders University, Adelaide, Australia). Manometric variables were extracted in accordance with the standardized protocol recommended by the Pharyngeal High‐Resolution Manometry Working Group [6]. The primary outcome measures included three groups of metrics: metrics of UES relaxation and metrics of UES contraction, metrics of pharyngeal constrictor muscle and proximal esophagus contraction, as illustrated in Figures 1 and 2 [8].

UES relaxation was quantified as intrabolus distension pressure (IBP, mmHg), integrated relaxation pressure (UESIRP, mmHg), relaxation time (UESRT, seconds), and the maximum admittance of the UES distension area (UES MaxAd, mS).

UES contraction was quantified as pre‐deglutitive UES basal pressure (UESBP, mmHg), post‐deglutitive UES peak pressure (UES PeakP, mmHg), and post‐deglutitive UES contractile integral (UESCI, mmHg cm s).

Pharyngeal constrictor muscle and proximal esophagus contraction were quantified using the total pharyngeal contractile integral (PhCI, mmHg cm s), as well as regional contractile integrals specific to the velopharyngeal (VCI), mesopharyngeal (MCI), and hypopharyngeal (HPCI) segments, all expressed in mmHg cm s. Additional variables included the hypopharyngeal peak pressure (HP PeakP, mmHg) and the proximal esophageal contractile integral (PCIes, mmHg).

### 2.4 Statistical Analysis

All data were analyzed using IBM SPSS Statistics version 22.0 and are expressed as means. Comparisons between placebo and mosapride conditions were performed using Student's *t* test. To analyze the effects of bolus volume and viscosity, a general linear model with repeated measures analysis of variance (ANOVA) was conducted. Treatment condition (mosapride vs. placebo) and bolus viscosity (IDDSI Level 0 vs. 4) were treated as between‐subject categorical variables, while bolus volume (5, 10, and 20 mL) was treated as a within‐subject factor. When the assumption of sphericity was violated, Greenhouse–Geisser correction was applied. A *p* < 0.05 was considered statistically significant. Univariate within‐subject effects are reported.

## 3 Results

Twenty‐four volunteers (13 males, 11 females; mean age 33 years; range 24–56 years) were enrolled in this study. All participants completed both sessions of the study protocol without protocol violations or reported adverse effects. No participants were excluded. Prior to the initial manometric assessment, participants were randomized into two groups as 15 received mosapride and nine received placebo. There were no statistically significant differences in baseline characteristics between the groups. Specifically, the mean age was 40 years in the mosapride‐first group and 33 years in the placebo‐first group (*p* = 0.272). Gender distribution was also comparable, with eight males and seven females in the mosapride‐first group and 5 males and 4 females in the placebo‐first group (*p* = 0.920).

### 3.1 UES Relaxation Metrics

Mosapride did not alter UES relaxation metrics (Tables 1 and 2). While unadjusted comparisons using the Student's *t* test suggested that UESIRP (5 mL *p* = 0.017, 10 mL *p* = 0.008) and UES maximal opening admittance (UES MaxAd; 10 mL *p* = 0.018, 20 mL *p* = 0.017) were significantly altered during thick swallows under mosapride exposure (Table 1), these differences did not remain statistically significant following ANOVA adjustment (Table 2).

**TABLE 1:** Upper esophageal sphincter (UES) metrics during thin (IDDSI Level 0) and thick (IDDSI Level 4) liquid swallows in volunteers following mosapride or placebo administration.

	Volume	Mosapride mean (95% CI)	Placebo mean (95% CI)	*p*
UESIRP	*IDDSI 0*			
	5 mL	7.41 (5.00–9.81)	7.93 (4.83–11.03)	0.365
	10 mL	7.17 (5.03–9.31)	7.47 (5.14–9.79)	0.301
	20 mL	8.35 (6.33–10.37)	8.20 (5.80–10.59)	0.204
	*IDDSI 4*			
	5 mL	7.72 (5.94–9.50)	9.72 (7.27–12.16)	0.017*
	10 mL	9.82 (8.34–11.29)	11.54 (8.99–14.08)	0.008*
	20 mL	12.86 (10.91–14.81)	13.92 (11.56–16.28)	0.277
UESRT	*IDDSI 0*			
	5 mL	0.47 (0.44–0.51)	0.47 (0.42–0.51)	0.828
	10 mL	0.47 (0.43–0.51)	0.48 (0.44–0.51)	0.925
	20 mL	0.52 (0.47–0.58)	0.52 (0.47–0.56)	0.846
	*IDDSI 4*			
	5 mL	0.46 (0.40–0.52)	0.44 (0.40–0.47)	0.511
	10 mL	0.49 (0.43–0.54)	0.45 (0.41–0.49)	0.254
	20 mL	0.53 (0.48–0.59)	0.53 (0.46–0.59)	0.820
UESMaxAd	*IDDSI 0*			
	5 mL	3.68 (3.44–3.92)	3.65 (3.38–3.93)	0.856
	10 mL	4.60 (4.25–4.95)	4.57 (4.24–4.90)	0.711
	20 mL	4.82 (4.41–5.23)	5.22 (4.87–5.57)	0.948
	*IDDSI 4*			
	5 mL	3.99 (3.71–4.28)	3.81 (3.36–4.27)	0.053
	10 mL	4.77 (4.43–5.12)	4.37 (3.75–4.99)	0.018*
	20 mL	5.53 (5.12–5.95)	5.04 (4.25–5.83)	0.017*
UESBP	*IDDSI 0*			
	5 mL	48.46 (42.70–54.23)	56.00 (48.74–63.27)	0.100
	10 mL	50.03 (44.06–56.00)	56.54 (46.89–66.19)	0.241
	20 mL	52.63 (46.75–58.51)	58.24 (51.57–64.92)	0.198
	*IDDSI 4*			
	5 mL	39.54 (35.72–43.37)	45.57 (40.71–50.43)	0.050
	10 mL	42.72 (37.58–47.87)	47.77 (41.64–53.91)	0.199
	20 mL	45.18 (39.30–51.05)	49.52 (43.61–55.43)	0.287
UESCI	*IDDSI 0*			
	5 mL	583.80 (486.67–680.94)	616.01 (541.22–690.79)	0.589
	10 mL	554.55 (477.23–631.86)	615.29 (539.70–690.87)	0.251
	20 mL	611.99 (547.34–676.65)	628.83 (560.84–696.83)	0.712
	*IDDSI 4*			
	5 mL	541.81 (475.80–607.83)	571.83 (504.84–638.81)	0.512
	10 mL	572.95 (501.09–644.80)	609.74 (548.55–670.93)	0.424
	20 mL	639.46 (554.14–724.78)	699.21 (623.76–774.66)	0.283
UES PeakP	*IDDSI 0*			
	5 mL	153.03 (136.36–169.69)	159.85 (143.62–176.07)	0.547
	10 mL	151.77 (135.57–167.97)	160.43 (143.32–177.55)	0.451
	20 mL	154.04 (138.89–169.19)	163.34 (145.09–181.58)	0.422
	*IDDSI 4*			
	5 mL	146.43 (131.25–161.61)	153.75 (137.31–170.19)	0.502
	10 mL	149.86 (134.55–165.17)	154.42 (139.14–169.70)	0.665
	20 mL	157.83 (141.34–174.32)	162.27 (146.50–178.04)	0.689

Abbreviations: CI, confidence interval; IDDSI, International Dysphagia Diet Standardization Initiative; UES, upper esophageal sphincter; UES MaxAd, upper esophageal sphincter distension area maximum admittance; UES PeakP, post‐deglutitive upper esophageal sphincter peak pressure; UESBP, pre‐deglutitive upper esophageal sphincter basal pressure; UESCI, post‐deglutitive upper esophageal sphincter contractile integral; UESIRP, upper esophageal sphincter integrated relaxation pressure; UESRT, upper esophageal sphincter relaxation time.

* *p* < 0.05.

**TABLE 2:** Between‐subject effects and interaction terms for swallowing metrics based on repeated measures ANOVA.

	Mosapride	Consistency	Mosapride × consistency
PhCI	0.319	0.500	0.873
VCI	0.840	0.125	0.797
MCI	0.319	0.500	0.873
HPCI	0.536	0.933	0.945
IBP	0.989	0.218	0.844
HP PeakP	0.846	0.409	0.930
UESIRP	0.370	0.002*	0.497
UESRT	0.547	0.804	0.654
UESMaxAd	0.525	0.397	0.217
UESBP	0.040*	0.003*	0.802
UESCI	0.233	0.901	0.932
UES PeakP	0.696	0.370	0.853
PCIes	0.156	0.844	0.822

*Note*: Mosapride × consistency: interaction between mosapride and consistency.

Abbreviations: HP PeakP, hypopharyngeal peak pressure; HPCI, hypopharyngeal contractile integral; IBP, intrabolus distension pressure; MCI, mesopharyngeal contractile integral; PCIes, proximal esophageal contractile integral; PhCI, pharyngeal contractile integral; UES, upper esophageal sphincter; UES MaxAd, upper esophageal sphincter distension area maximum admittance; UES PeakP, post‐deglutitive upper esophageal sphincter peak pressure; UESBP, pre‐deglutitive upper esophageal sphincter basal pressure; UESCI, post‐deglutitive upper esophageal sphincter contractile integral; UESIRP, upper esophageal sphincter integrated relaxation pressure; UESRT, upper esophageal sphincter relaxation time; VCI, velopharyngeal contractile integral.

* *p* < 0.05.

In terms of bolus characteristics, bolus volume exerted a significant main effect on IBP (*p* = 0.002), UESIRP (*p* < 0.001), UESRT (*p* < 0.001), UES MaxAd (*p* < 0.001, Table 3). Bolus consistency also had a significant main effect on UESIRP (*p* = 0.002; Table 2). Additionally, there was a significant interaction between bolus volume and viscosity in relation to UESIRP (*p* < 0.001; Table 3). No significant interactions were detected between mosapride and bolus volume or between mosapride and bolus consistency for any UES relaxation parameters (Tables 2 and 3).

**TABLE 3:** Within‐subject effects and interaction terms for swallowing metrics based on repeated measures ANOVA.

	Volume	Volume × consistency	Volume × mosapride	Volume × consistency × mosapride
PhCI	< 0.001*	0.325	0.321	0.514
VCI	< 0.001*	0.347	0.307	0.784
MCI	< 0.001*	0.325	0.321	0.514
HPCI	0.315	0.017*	0.654	0.606
IBP	0.002*	0.696	0.163	0.749
HP PeakP	0.174	0.840	0.778	0.328
UESIRP	< 0.001*	< 0.001*	0.535	0.966
UESRT	< 0.001*	0.167	0.777	0.419
UESMaxAd	< 0.001*	0.139	0.484	0.057
UESBP	< 0.001*	0.614	0.630	0.998
UESCI	< 0.001*	0.002*	0.755	0.393
UES PeakP	0.001*	0.068	0.978	0.628
PCIes	< 0.001*	0.012*	0.701	0.998

*Note*: Volume × consistency: interaction between volume and consistency; volume × mosapride: interaction between volume and mosapride; volume × consistency × mosapride, interaction among volume, consistency and mosapride.

Abbreviations: HP PeakP, hypopharyngeal peak pressure; HPCI, hypopharyngeal contractile integral; IBP, intrabolus distension pressure; MCI, mesopharyngeal contractile integral; PCIes, proximal esophageal contractile integral; PhCI, pharyngeal contractile integral; UES, upper esophageal sphincter; UES MaxAd, upper esophageal sphincter distension area maximum admittance; UES PeakP, post‐deglutitive upper esophageal sphincter peak pressure; UESBP, pre‐deglutitive upper esophageal sphincter basal pressure; UESCI, post‐deglutitive upper esophageal sphincter contractile integral; UESIRP, upper esophageal sphincter integrated relaxation pressure; UESRT, upper esophageal sphincter relaxation time; VCI, velopharyngeal contractile integral.

* *p* < 0.05.

### 3.2 UES Contraction Metrics

Mosapride did not alter most UES contraction metrics except UESBP (Tables 1 and 2). During 5 mL thick swallows, a trend toward reduced UESBP with mosapride administration was observed, reaching marginal statistical significance (39.54 vs. 45.57 mmHg; *p* = 0.050; Table 1). However, this effect was not seen with other bolus volumes or during thin consistency swallows (Table 1). After adjusting for the effects of bolus volume and viscosity using repeated measures ANOVA, the overall effect of mosapride on UESBP reached statistical significance (*p* = 0.040; Table 2).

In terms of bolus characteristics, bolus volume exerted a significant main effect on pre‐deglutitive UESBP (*p* < 0.001), post‐deglutitive UESCI (*p* < 0.001), post‐deglutitive UES PeakP (*p* = 0.001; Table 3). Bolus consistency also had a significant main effect on pre‐deglutitive UESBP (*p* = 0.003; Table 2). Additionally, there was a significant interaction between bolus volume and viscosity in relation to post‐deglutitive UESCI (*p* = 0.002; Table 3). No significant interactions were detected between mosapride and bolus volume, or between mosapride and bolus consistency for any UES contraction metrics (Tables 2 and 3).

### 3.3 Pharyngeal Constrictor Muscle and Proximal Esophagus Contraction Metrics

Mosapride administration did not exert a significant overall effect on pharyngeal constrictors muscle and proximal esophagus contraction variables (Table 2). Bolus volume was found to significantly influence the total PhCI (*p* < 0.001), VCI (*p* < 0.001), mesopharyngeal contractile integral (MCI; *p* < 0.001), and PCIes (*p* < 0.001; Table 3). A significant interaction between bolus volume and bolus viscosity was observed in relation to the HPCI (*p* = 0.017) and PCIes (*p* < 0.001; Table 3). No significant interactions were found between mosapride and bolus volume for any of the pharyngeal constrictor muscle and proximal esophagus contraction metrics (Table 3).

## 4 Discussion

This exploratory, placebo‐controlled study investigated the acute effects of mosapride on swallowing physiology in individuals with no history or symptoms of dysphagia. The main finding was that mosapride administration led to a reduction in UESBP, an effect that was more pronounced during the swallowing of viscous boluses. No significant effects were observed on other parameters of pharyngeal peristalsis.

These results suggest a potential influence of mosapride, a 5‐HT4 receptor agonist, on oropharyngeal motor function. Mosapride is widely prescribed in clinical practice for its prokinetic effects on upper gastrointestinal motility. Although indirect effects of mosapride, such as a reduced risk of aspiration due to enhanced gastric emptying, have been postulated [9]. The direct impact of mosapride on pharyngoesophageal physiology has not been previously investigated.

In our study, mosapride appeared to attenuate UES basal contractility. Given the limited data regarding 5‐HT4 receptor involvement in striated muscle activity, the mechanisms underlying this observation remain speculative. However, 5‐HT4 receptors are known to be distributed within the central nervous system, particularly in subcortical regions such as the basal ganglia, which play a key role in the neural circuitry of volitional swallowing during the preparatory phase [10, 11, 12]. It is therefore plausible that mosapride's influence on UES basal tone is mediated through modulation of central motor pathways involving the basal ganglia.

Despite the observed change in basal tone, our study did not identify a significant acute effect of mosapride on UES relaxation metrics. Nonetheless, previous clinical studies have suggested that chronic administration of mosapride (e.g., 5 mg prior to meals) may reduce aspiration events and associated complications in patients with post‐stroke dysphagia, potentially through indirect mechanisms such as improved gastric emptying and reduced reflux burden [9]. Whether such chronic administration exerts cumulative or adaptive effects on swallowing dynamics remains unknown and warrants further investigation.

There were several limitations to our study. First, the small sample size limited statistical power and the generalizability of the findings. Second, the open‐label study design may introduce bias related to participant or investigator expectations. Third, our investigation was limited to acute exposure and therefore cannot provide conclusions regarding the long‐term effects of mosapride on swallowing function.

In conclusion, mosapride appears to influence pharyngeal swallowing by reducing UES basal tone, suggesting a potential neurological mechanism involving 5‐HT4 receptor‐mediated modulation of UES contractility. While the drug did not acutely affect most pharyngeal peristaltic parameters, its impact on basal UES pressure may have clinical implications, particularly in conditions where UES tone is pathologically elevated. Given the physiological role of the UES in preventing pharyngeal reflux of gastric contents, further studies are warranted to determine whether mosapride modulates pharyngeal reflux and aspiration risk, especially with prolonged use.

## Conflicts of Interest

T. Omari holds inventorship of the international patent family that covers the analytical methods described. The Swallow Gateway software service is owned and provided by Flinders University. The other authors declare no conflicts of interest.

## Data Availability

The data that support the findings of this study are available from the corresponding author upon reasonable request.
